# Symbiosis of intracellular bacteria Wolbachia with insects: a hundred years of study summarized

**DOI:** 10.18699/vjgb-25-10

**Published:** 2025-02

**Authors:** O.D. Shishkina, N.E. Gruntenko

**Affiliations:** Institute of Cytology and Genetics of the Siberian Branch of the Russian Academy of Sciences, Novosibirsk, Russia; Institute of Cytology and Genetics of the Siberian Branch of the Russian Academy of Sciences, Novosibirsk, Russia

**Keywords:** Wolbachia, insects, Drosophila melanogaster, Wolbachia, насекомые, Drosophila melanogaster

## Abstract

Wolbachia pipientis is an α-proteobacterium, which is a widespread intracellular symbiont in a number of Arthropoda and some Nematoda species. With insects, W. pipientis forms a symbiont-host system characterized by very close interactions between its components. The mutual effects of Wolbachia on the host and the host on Wolbachia are important biotic factors for both components of this symbiotic system. Wolbachia is able to affect both host reproduction and somatic organ function. Due to its prevalence among insects and a wide variety of both negative (cytoplasmic incompatibility and androcide are among the most well-known examples) and positive (increasing resistance to biotic and abiotic factors, providing vitamins and metabolites) effects on the host organism, Wolbachia is of great interest for both entomologists and microbiologists. The diversity of host phenotypes induced by Wolbachia provides a broad choice of evolutionary strategies (such as reproductive parasitism or mutually beneficial symbiont-host relationships) that it utilizes. The influence of Wolbachia is to be considered in the design of any experiment conducted on insects. The application of sequencing technologies has led to new approaches being created to study the existing relationships within the Wolbachia-insect system, but interpretation of the data obtained is challenging. Nevertheless, the prospects for the use of the whole-genome analysis data to study Wolbachia-host coevolution are beyond doubt. Ongoing projects to introduce Wolbachia strains, which provide antiviral host defense, into insect populations to control the spread of RNA-viruses are actively pursued, which could result in saving many human lives. The aim of this brief review is to summarize the data collected by scientists over the past hundred years of Wolbachia studies and the current understanding of its genetic diversity and mechanisms of interaction with the host, including those based on transcriptome analysis.

## Abstract (ru)

Wolbachia pipientis – α-протеобактерия, широко распространенный внутриклеточный симбионт у ряда видов Arthropoda и некоторых видов Nematoda. Вместе с насекомыми W. pipientis образует систему «симбионт–хозяин», характеризующуюся очень тесными взаимодействиями между ее компонентами. Влияния в обоих направлениях, которые оказывает как вольбахия на хозяина, так и хозяин на вольбахию, являются важными биотическими факторами для обеих составляющих этой симбиотической системы. Вольбахия способна оказывать воздействие как на размножение хозяина, так и на работу соматических органов. Благодаря своей распространенности среди насекомых и большому разнообразию как отрицательных (среди самых известных примеров – цитоплазматическая несовместимость и андроцид), так и положительных эффектов (повышение устойчивости к биотическим и абиотическим факторам, обеспечение витаминами и метаболитами), оказываемых на организм хозяина, вольбахия вызывает огромный интерес у энтомологов и микробиологов. Разнообразие вызываемых вольбахией фенотипов хозяина обеспечивает широкий выбор эволюционных стратегий, таких как репродуктивный паразитизм или взаимовыгодные отношения между симбионтом и хозяином, которыми она пользуется. Влияние вольбахии необходимо учитывать при постановке любого эксперимента, проводимого на насекомых. Применение технологий секвенирования привело к появлению новых подходов для изучения существующих связей внутри системы «Wolbachia–насекомое», однако интерпретация полученных данных представляет определенную сложность. Тем не менее перспективы использования данных полногеномного анализа для изучения коэволюции Wolbachia и хозяина не вызывают сомнений. Активно осуществляются проекты по внедрению в популяции насекомых штаммов вольбахии, обеспечивающих противовирусную защиту хозяина, для контроля распространения РНК-вирусов, что может способствовать спасению многих человеческих жизней. Целью этого обзора стало обобщение данных, полученных учеными за прошедшие сто лет изучения Wolbachia, и современных представлений о ее генетическом разнообразии и механизмах взаимодействия с хозяином, в том числе основанных на данных транскриптомного анализа.

## Introduction

Relations within the endosymbiont-host system deserve considerable attention from an evolutionary perspective because mutual adaptations of the symbiont to the host and the host to the symbiont guide the advancement of both species. Despite that, numerous surprising effects of symbiont influence on the host were not immediately linked to infection status. The observed effects of the intracellular α-proteobacterium Wolbachia on host insects are particularly well documented, but even in this symbiotic system the relationships remain poorly understood. At present, numerous studies of specific Wolbachia strains and their impact on completely different aspects of host species are being conducted using wholegenome sequencing and transcriptomic analysis. The purpose of this brief review is to highlight the progress that has been made in the field of studying the Wolbachia-host symbiotic system.

## The establishment and development of an interest in Wolbachia

The genus Wolbachia belongs to the family Anaplasmataceae, a member of the order Rickettsiales, class α-proteobacteria (Hertig, Wolbach, 1924). Wolbachia is a widespread intracellular symbiont bacterium of a number of Arthropoda species and some Nematoda species. Approximately 50 % of all insect species on our planet are infected with this bacterium (Hilgenboecker et al., 2008; Zug, Hammerstein, 2012). The estimations of different groups of researchers vary due to the difficulty of conducting such large-scale studies and limitations in sample sizes. There is variation in the frequency of infection in different geographical locations, and in some, infection occurs at very low frequencies, which increases the likelihood of false negatives when testing for Wolbachia (since there is an increased probability of randomly selecting a sample without Wolbachia, even though it occurs in the host population).

Although the discovery of this bacterium took place a century ago (Hertig, Wolbach, 1924), even the specification of the number of species in the genus Wolbachia has been a matter of debate for many decades. The reason is that there is no clear concept of species boundaries applicable to endosymbiotic bacteria. At the moment, it is accepted that all discovered variants of Wolbachia belong to one species, Wolbachia pipientis. In this paper, according to tradition, this bacterium will be referred to as Wolbachia (genus name only) or Wolbachia pipientis (name of the species that has remained traditionally). However, it should be noted that there is still no established consensus in the research community on the vagueness of the taxonomy of this genus (Lo et al., 2007).

It is believed that M. Hertig and S.B. Wolbach (1924) were driven to the discovery of the bacterium, which has been defined as “rickettsia-like,” by a deadly typhoid epidemic (Porter, Sullivan, 2023). Typhus is a disease, the source of which is the bacterium Rickettsia prowazekii, a bacterium carried by the body louse Pediculus humanus corporis (Linnaeus, 1758). As a result of the search for potential agents of typhus, other intracellular organisms have been discovered that later acquired the name Wolbachia pipientis (Porter, Sullivan, 2023). Although Wolbachia is not a threat to humans, as with many discoveries in biology, the initial stimulus for the development of the study of this genus came from medicine.

After the first discovery of this bacterium and several years of dormancy, the next discovery that revitalized interest in Wolbachia was the conditional sterility of some insects caused by certain Wolbachia strains. To this day, this effect is the most well-known when it comes to this bacterium (Burdina, Gruntenko, 2022). The underlying mechanism behind this phenomenon is called cytoplasmic incompatibility (CI) (Laven, 1967). The way cytoplasmic incompatibility is realized in the first mitotic division of the zygote was later studied cytologically (Ryan, Saul, 1968). But only a relatively short time ago the elements that cause CI have been elucidated (Beckmann et al., 2017; LePage et al., 2017; Chen et al., 2019).

The current understanding of the prevalence of Wolbachia in insects would not be possible without the PCR identification of Wolbachia-specific DNA-markers. Even with the latest light and fluorescence microscopes, it is difficult to repeat M. Hertig and S.B. Wolbach’s achievement (Hertig, Wolbach, 1924) for other insects because Wolbachia are often inferior in size (diameter 0.25 to 1.8 μm) even to mitochondria (Yu, Walker, 2006). Screening as many insect species as possible by analyzing cytological specimens, which for each host species requires several specimens isolated from populations (single isolates), is an almost impossible task, while the same volume of isolates examined by the more sensitive PCR method requires less time and effort. With the help of this key molecular technique, modern biology has been able to discover that Wolbachia lives in almost all insects on the planet (Hilgenboecker et al., 2008).

## Influence of Wolbachia on the host

Wolbachia are vertically transmitted from the mother to the offspring through the cytoplasm of oocytes. The transmission mechanism may not always run flawlessly and sometimes spontaneous loss of infection occurs (Werren, 1997). Nevertheless, Wolbachia is consistently found in natural and laboratory insect populations. Wolbachia has no free-living analogues; all representatives of the Rickettsiales order, to which it belongs, are intracellular organisms (Yu, Walker, 2006). The ecological niche occupied by Wolbachia is the internal environment of its animal host. It grows in the cytoplasm of its host cell in the membrane-bound vacuole (Yu, Walker, 2006). For that reason the interactions occurring between Wolbachia and the host are very intimate and are both mutualistic and parasitic in nature (Burdina, Gruntenko, 2022).

Maternal inheritance is a common feature for mitochondria and Wolbachia. In addition, they are inherited in a linked manner rather than independently, forming a certain “cytotype” (Ilinsky, 2013). Due to the intracellular nature of this bacterium, its study is complicated; for example, it is difficult to explore the specifics of its metabolism. The establishment of passaged cell cultures of the insect hosts containing Wolbachia is possible, but also complicated. The first such cell line was created from cells of the mosquito Aedes albopictus (O’Neill et al., 1997). Stable cell cultures might become an invaluable tool for studying this genus, but this is made challenging by the spontaneous loss of infection that often occurs in them.

Wolbachia can only live in symbiosis with its host, but most host species are able to live and reproduce while uninfected. The influence Wolbachia has on the host is demonstrated in a number of different traits that can be observed when comparing infected host individuals with uninfected ones, as well as comparing individuals infected with different strains (Burdina, Gruntenko, 2022). The successful expansion of Wolbachia is partially explained by the ability of this organism to interfere with sex determination mechanisms, alter the development and reproductive patterns of the host for its own benefit

Among the numerous effects of Wolbachia, the most well-studied ones are those it has on the reproductive function of the host:

• androcide – selective death of males on the embryonic or larval developmental stage

• feminization of genetic males – acquisition of phenotypic traits of females by infected males

• stimulation of parthenogenesis

• cytoplasmic incompatibility

The most attention was always paid to the phenomenon of cytoplasmic incompatibility caused by Wolbachia. CI in insects is defined as follows: infected females can reproduce by being fertilized by both uninfected males and infected males, while uninfected females cannot reproduce with infected males (Kaur et al., 2021). Thus, infected females do not experience the negative consequences of CI and have reproductive advantage. Since Wolbachia is inherited through the maternal lineage along with the cytoplasm, this mechanism ensures that Wolbachia is effectively spread in insect populations (Lassya, Karrb, 1996).

Molecular mechanisms responsible for causing CI are connected to the disruption of the first mitotic division of the zygote (Poinsot et al., 2003). It has been shown, that the deubiquitylase CidA, which initiates CI in males, and protein CidB, which allows to overcome it, when expressed in females, are involved in the formation of CI (Beckmann et al., 2017). This confirms the previously formulated hypothesis of the “modification–rescue” pair put forward to explain the phenomenon of CI (Werren, 1997). Genes of the CI factors, called cifA–cifB pairs, were found to be integrated from the prophage WO into the genomes of Wolbachia that cause CI in the host (LePage et al., 2017). An alternative mechanism utilizes the nuclease CinA and its binding protein CinB (Chen et al., 2019), which also operate as the same “modifier– rescuer”. No other mechanism of Wolbachia’s influence on the host has been described in such detail.

Wolbachia-induced reproductive effects have been found in various insects, but there are exceptions, for example, they are completely not characteristic or weakly manifested in the most of the studied Drosophila melanogaster lines (Fry et al., 2004; Ilinsky, Zakharov, 2011).

**Fig. 1.**
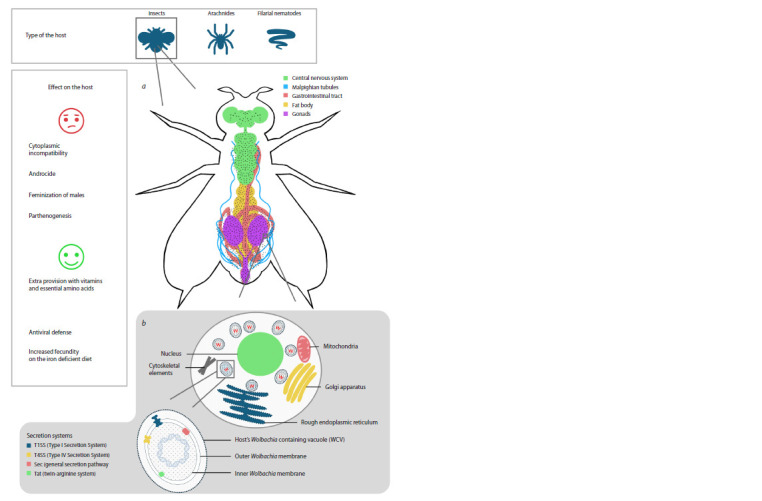
Scheme of Wolbachia–insect symbiotic system at different levels of organization а – localization of Wolbachia in different organs of Drosophila; b – localization of Wolbachia in the insect host cell and organelles that interact with Wolbachia. The Wolbachia-containing vacuole is shown at the bottom of the illustration

Besides Wolbachia’s influence on reproduction, numerous effects it causes on the somatic cells of the host have been discovered. This is possible due to the fact that Wolbachia is found not only in the reproductive organs of the females, where it is the most expected based on the mechanism of transmission of this symbiont to offspring, but also in the fat body, Malpighian vessels, muscle and nerve tissues (Fig. 1a) (Pietri et al., 2016). A variant of the pathogenic Wolbachia strain wMelPop, infecting D. melanogaster and known for causing premature death of the flies (Min, Benzer, 1997), wMelPop-CLA, changes male behavior, reducing male aggression by decreasing octopamine production in the brain (Rohrscheib et al., 2015). Individuals of D. melanogaster with the same nuclear genotype but infected with different strains of Wolbachia have different optimal temperature ranges (Truitt et al., 2019). This may affect the prevalence of certain strains at different latitudes.

Maintaining an endosymbiont is usually associated with costs to the host in terms of resources that both it and the bacterium require. Often more successfully selected by evolution are those symbionts that can provide greater benefit to the host. This minimizes the effect of its costs in maintaining the symbiont, and a mutually beneficial relationship is established in the system. The strategy of providing benefit by positively affecting aspects of the host’s life may explain why Wolbachia is so common among species in which its manipulation of the host’s reproductive system is not pronounced.

Increased lifespan has been shown in infected Drosophila (Maistrenko et al., 2016); Wolbachia can also supply their hosts with vitamins and essential amino acids. For example, the wCle strain, infecting the bedbug Cimex lectularius, provides the host with vitamin B7 (biotin) (Newton, Rice, 2020). Wolbachia protects the cells of parasitoid wasps Asobara tabida from iron excess through expression of bacterioferritin (Kremer et al., 2009), and in D. melanogaster infected with Wolbachia, fecundity is increased when maintained on iron-deficient diet (Brownlie et al., 2009).

Bacterioferritin binds free divalent iron and promotes absorption in the gut of the fly larvae (Brownlie et al., 2009). Evidence has been received that host insulin/insulinlike growth factor signaling cascade is suppressed and the hypoxia-inducible factor (HIF) signaling cascade is activated upon Wolbachia infection (Currin-Ross et al., 2021). Wolbachia have been shown to require iron acquisition from the host, based on which the authors suggest that iron is a fundamental aspect of Wolbachia–host interactions (Currin- Ross et al., 2021).

Because reproductive anomalies induced by Wolbachia are not characteristic of D. melanogaster, other physiological effects observed in this symbiotic pair have received more attention. There have been numerous studies carried out on the effects of specific strains of this bacterium on different lines of this host species. This makes Wolbachia–D. melanogaster symbiotic system one of the most studied in terms of the genetic diversity of both the host and the bacterium, as well as the effect of the combination of their genotypes on host fitness.

For instance, three lines of D. simulans of different origin, but infected with the same Wolbachia strain, showed different effects of the symbiont on host adaptability: in one of the lines under study, the introduction of Wolbachia by microinjection increased the fitness estimated in population cage assays, meanwhile, in two other lines, the fitness was not influenced by the bacterium (Dean, 2006). Different effects of Wolbachia on the lifespan, fecundity and developmental rate of different D. melanogaster lines were also found by A.J. Fry and D.M. Rand (2002) and N.V. Adonyeva et al. (2023).

On the other hand, infecting a single line of D. melanogaster with different Wolbachia variants resulted in changes in dopamine metabolism in flies infected with Wolbachia of the wMelCS genotype, but not in those infected with Wolbachia of the wMel genotype (Gruntenko et al., 2017; Burdina et al., 2021). Similar differences in the influence of Wolbachia genotype on its effects on host physiology have been shown for juvenile hormone metabolism (Gruntenko et al., 2019).

At the same time, several effects on D. melanogaster attributed to Wolbachia, as far as is currently known, do not depend on symbiont genotype. So, infection of one line of D. melanogaster with seven different Wolbachia variants promoted an increase in the host fly’s lipid stores (Karpova et al., 2023). An increase in glucose and triglyceride (TAG) content in the host was also common to different bacterial variants (Zhang et al., 2021; Karpova et al., 2023), but trehalose levels remained unchanged in all lines compared to uninfected flies (Karpova et al., 2023). These lines differed from uninfected lines in their increased survival under nutritional deficiency. Increased glucose-6-phosphate levels were also observed in Wolbachia-infected mosquitos Aedes fluviatilis (da Rocha Fernandes et al., 2014).

A study carried out on a transgenic line of D. melanogaster with impaired function of insulin receptor showed that the presence of Wolbachia increases the adaptability of such mutants (Ikeya et al., 2009). Removal of Wolbachia by antibiotics in such flies resulted in an enhanced mutant phenotype (which is manifested by reduced growth and fecundity). The authors hypothesized that Wolbachia activates insulin/insulin-like growth factor (I/IGF) signaling cascade (Ikeya et al., 2009). However, a more recent study suggests otherwise. In the work (Currin-Ross et al., 2021), they examined the metabolic response of D. melanogaster to infection status and showed that the I/IGF-mediated signaling pathway is suppressed by Wolbachia.

Some strains of Wolbachia are known to improve the host’s defense against a number of pathogens, as they are able to inhibit the replication of RNA viruses (Hedges et al., 2008; Teixeira et al., 2008; Moreira et al., 2009). Due to their antiviral defense properties, Wolbachia are used for biological control purposes (Hoffmann et al., 2011; LePage, Bordenstein, 2013). A number of Wolbachia strains, the native host of which is D. melanogaster, have been introduced by microinjection into individuals of the mosquito Aedes aegypti, which is a vector of dengue virus (dengue virus – DENV) (Hoffmann et al., 2011, Gu et al., 2022). Introduction of Wolbachia-infected individuals into natural populations resulted in their successful spread due to CI (Hoffmann et al., 2011), which may reduce the efficiency of dengue virus transmission, since blocking of the latter by Wolbachia in Ae. aegypti has been demonstrated in laboratory conditions (Gu et al., 2022).

There are several hypotheses as to how different properties of Wolbachia strains may influence antiviral defense, and selection of the most effective strains is the goal of many studies. Since CI promotes the predominant spread of a particular strain (the one that causes this abnormality in the host) in the population, the joint inheritance of antiviral defense and the ability to induce CI makes such strains more effective when using a substitution strategy. It is noted that strains characterized by increased Wolbachia content in host cells (such as wMelPop) contribute more to the host’s ability to successfully fight the virus. Based on this fact, it is hypothesized that there is a correlation between the effectiveness of antiviral defense and high Wolbachia content in cells (Chrostek et al., 2013; Gu et al., 2022). However, the optimal temperature range for Wolbachia strains in the habitat of the insects, into the population of which a new Wolbachia strain is introduced, is also worth considering. Attempts have been made to use Wolbachia to control other arboviruses that pose a threat to humans (Kamtchum-Tatuene et al., 2017).

The adaptive or deleterious nature of some Wolbachia effects is difficult to determine unequivocally, but it is generally clear that some of them (for example, manipulation of host reproduction) can be attributed to parasitic effects, whereas other effects, such as increased resistance to viral infection and starvation, provide an adaptive advantage not only to endosymbionts in this system but also to host insects. The full range of effects of Wolbachia on the host cannot be considered without addressing the diversity of strains of this bacterium, as many of the effects it exerts are specific to a particular strain of Wolbachia. Although Wolbachia genomes share a common core set of genes, different strains differ significantly from each other.

## Genetic diversity of Wolbachia

Since it is generally accepted that there is only one species of Wolbachia – W. pipientis (Hertig, Wolbach, 1924), the entire diversity of these insect endosymbionts is described by different strains divided into supergroups. The division into supergroups is based on phylogenetic analysis of the sequences of several genes used for multilocus typing. Several groups of genes for multilocus typing of Wolbachia strains have been proposed: dnaA, 16SrRNA, wsp, gltA and groEL, ftsZ (Lo, Evans, 2007), gatB, hcpA, fbpA, coxA (Baldo et al., 2006b). According to different sources, from 10 to 13 supergroups are distinguished, designated by Latin letters A–F, N–M and S (Kaur et al., 2021); the classical classification includes seven supergroups (A–F and H) (Ros et al., 2009; Augustinos et al., 2011).

The most universal genotyping system – the process of identifying genetic differences and similarities between different groups of organisms – for Wolbachia strains is currently multilocus sequence typing (MLST), which uses five protein-coding genes: ftsZ, gatB, coxA, hcpA and fbpA (Baldo et al., 2006b). Based on analysis of the combination of 5 or more polymorphic markers, ST (sequence type) profiles are compiled. The utilization of several alleles as markers provides more accurate and complete information than the utilization of a single allele.

The genomes of Wolbachia are characterized by a wide diversity, which is also formed by strain isolation due to maternal inheritance along with the cytoplasm. Although the hosts may be closely related species, their associated Wolbachia strains can differ greatly at the genetic level. Sequences from hypervariable loci can be used to separate recently diverged strains, although the possibility of recombination of Wolbachia strains, which has been demonstrated experimentally (Baldo et al., 2006a), and the presence of a large number of repeats and mobile elements in the Wolbachia genome (Wu et al., 2004) must be taken into account.

In the vast array of host–Wolbachia combinations, each is characterized by its own unique set of adaptations of the symbiont to the host and vice versa, which affects the type of symbiotic relationship. In addition, new strains of this bacterium are discovered and described almost every year, and, as a general rule, researchers focus their work on the effects of specific Wolbachia strains on their objects of interest (Burdina et al., 2021; Duarte et al., 2021; Ilinsky et al., 2022).

We will examine in more detail the diversity of Wolbachia strains found in the classical model object D. melanogaster. Wolbachia infection in D. melanogaster was first detected in 1988 (Hoffman, 1988), but the wMel strain was described only ten years later (Zhou et al., 1998). In 2005, M. Riegler et al. (2005) identified five different Wolbachia genotypes in D. melanogaster based on polymorphic markers. Several different lineages were assumed to have originated from a single ancestral Wolbachia infection (Riegler et al., 2005; Hilgenboecker et al., 2008). In the literature, new and first described Wolbachia in D. melanogaster are usually referred to as strains (Lo et al., 2007). Often there is insufficient information in a study presenting a new strain to assign it to one of the known genotypes.

**Fig. 2.**
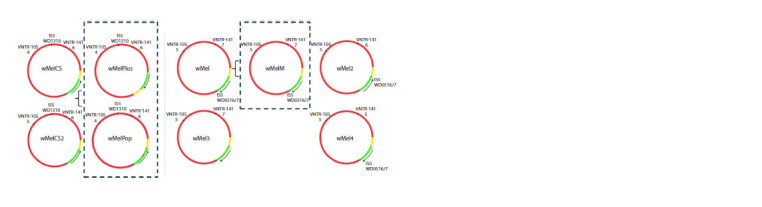
Chromosome maps of six different W. pipientis genotypes isolated from D. melanogaster, as well as three unique strains (wMelPlus and wMelPop, belonging to the wMelCS genotype, and wMelM, belonging to the wMel genotype). The green color indicates the inversion that distinguishes the wMel genotype from wMelCS. The yellow and blue regions denote sequences included in the inversion in wMelPlus but unaffected by the inversion in the wMel group. The magenta region denotes the Octomom sequence (Chrostek, Teixeira, 2018).

To date, six genotypes of W. pipientis found in D. melanogaster have been described (Fig. 2). They are divided into two groups: wMel (which includes genotypes wMel, wMel2, wMel3, wMel4) and wMelCS (which includes wMelCS and wMelCS2) (Riegler et al., 2005; Ilinsky, 2013). Sequencing of Wolbachia genomes revealed the presence of a large number of repeats, including insertion sequences (IS) and variable number tandem repeats (VNTR). Genotypes are distinguished by polymorphisms of five genome markers: the presence of inversion in the locus WD0394-WD0541 (in Figure 2, the direction of the fragment is indicated by an arrow); variable number tandem repeat markers VNTR- 105, VNTR-141 (in Figure 2, the number of repeats is indicated by numbers under them); IS5 WD1310, IS5 WD0516/7 – IS element insertion loci. These markers are used for genotyping Wolbachia from isolates of natural and laboratory populations of D. melanogaster (Riegler et al., 2005; Ilinsky, 2013).

It should be noted that two strains have also been described for the wMelCS genotype that differ in their effect on the host and in their genetic composition, although these differences are not detected by Riegler genotyping (Riegler et al., 2005). The first of these strains is the pathogenic strain wMelPop (from the word “popcorn”), which causes premature death of flies infected with it through its unrestricted proliferation leading to overcrowding and rupture of host cells (Min, Benzer, 1997) and has an increased copy number of a region of eight Octomom genes that has been associated with the pathology caused by the wMelPop strain (Chrostek et al., 2013; Chrostek, Teixeira, 2015). The second strain, wMelPlus (from “plus”, meaning a “positive sign”), not defined by M. Rigler et al. but distinguished by a large (approximately 1/6 of the genome) inversion from other representatives of the wMelCS genotype (Korenskaia et al., 2022), on the contrary, has a positive effect on host fitness, increasing its resistance to heat stress (Burdina et al., 2021). The discoveries of these strains were a great surprise when investigating the phenotypic differences between D. melanogaster lines carrying them and lines with “normal” characteristics. A strain named wMelM that increases host resistance of D. melanogaster to heat stress, but does not differ in markers (according to M. Rigler et al.) from the wMel genotype was also discovered (Gu et al., 2022). These three examples demonstrate that great genetic diversity can be hidden from researchers behind identical genotype labels

Whole-genome sequencing is suitable for detecting such differences in the genome of strains. It should be taken into account that when assembling the genome using a reference genome, it is possible to miss the presence of inversions (there are difficulties due to the presence of repeats in the genome and short lengths of reads while sequencing).

A number of issues related to Wolbachia genotypes that infect D. melanogaster deserve special attention. In natural populations of D. melanogaster, genotypes wMel and wMelCS are the most commonly found, with wMel significantly predominating (Riegler et al., 2005; Nunes et al., 2008; Ilinsky, 2013). It is hypothesized that this genotype gradually displaced the previously predominant wMelCS (Riegler et al., 2005). It has been shown that the effect of shifting thermal preference toward lower temperatures in Drosophila infected with Wolbachia compared to uninfected flies is strongest in D. melanogaster lines infected with Wolbachia strains of the wMelCS group (Truitt et al., 2019). On the other hand, there is evidence of low genetic polymorphism of wMelCS group genotypes in the Palaearctic, contradicting the hypothesis that the global replacement of Wolbachia genotypes occurred recently and indicating that there is still much that remains unknown in this field (Bykov et al., 2019).

The strain wMel is the first strain of the Wolbachia bacterium with a completely sequenced and annotated genome (Wu et al., 2004). The genome size of this strain is 1,267,782 bp; it includes about 1,270 protein-coding genes (Porter, Sullivan, 2023). No significant differences in size and gene composition from strains of the wMelCS genotype (excluding the unique wMelPop strain, which has a special genome region formed by Octomom sequence repeats (Duarte et al., 2021)) have been shown (Chrostek et al., 2013; Korenskaia et al., 2022).

## Studies dedicated to the mechanisms of interactions in the Wolbachia–host system

Large-scale searches for possible effector molecules, which Wolbachia can utilize to have an influence on the host’s organism, have been conducted (Ote et al., 2016; Sheehan et al., 2016; Rice et al., 2017). For a bacterium to influence processes within eukaryotic host’s cells, the effector molecules presumably must have homology with some molecules synthesized in the host organism.

The bacterial genome often acquires foreign genetic material from eukaryotic cells that retains at least some of its original activity, and the products of these domains are released into the cytoplasm of the eukaryotic cell (De Felipe et al., 2005). A study was conducted in which 163 gene candidates from the genome of the wMel strain were selected via bioinformatics methods, and then 84 transcription products of these genes were analyzed for their effects on the yeast Saccharomyces cerevisiae (Rice et al., 2017). In this analysis, yeast growth defects and 14 possible effector genes were identified (Rice et al., 2017), three of which contain ankyrin repeats, which may indicate their involvement in proteinprotein interactions with their arthropod hosts

Since there is a barrier between endosymbiont and host organisms, specialized secretion systems are required to release effector molecules outside the bacterium. Bacterial secretion systems consist of protein complexes and are responsible for the passage of macromolecules through membranes. In bacteria, secretion is necessary for adaptation to environmental conditions and to enable pathogenicity in some bacteria. Due to Wolbachia being an endosymbiont, secretion system is an important tool for interactions with the host’s cells. Wolbachia utilizes two types of secretion systems (Fig. 1b): T1SS – type I secretion system and T4SS – type IV secretion system (Lindsey, 2020). The first type of secretion system consists of three proteins: ABC-transporter, which is ATP-dependent, MFP – membrane fusion protein and OMP – outer membrane protein. The forth type of secretion system usually consists of 12 protein components: VirB1–VirB11 and VirD4 (Fronzes et al., 2009). Genes of this secretion system are located in two clusters in the Wolbachia genome: tandem genes of five proteins (VirB8, VirB9, VirB10, VirB11, VirD4) and those of three proteins (VirB3, VirB4, VirB6); meanwhile, genes VirB1, VirB2, VirB5 and VirB7 have been eliminated. The sequence and organization of these genes have been shown to be conserved in 37 Wolbachia strains under study (Pichon et al., 2009). These two secretory systems allow the secretion of a wide range of substrates, from single proteins to protein-protein and protein-DNA complexes (Backert, Meyer, 2006).

In the Wolbachia genomes, there are genes coding the channels of the Sec (general secretion system) and Tat (twin-arginine translocation) systems. These systems are involved in the protein transport through the Wolbachia’s cell membrane into the periplasmic space (Sec transports unfolded proteins, while Tat transports proteins folded to the tertiary structure) (Lindsey, 2020).

Wolbachia-containing vacuoles share a common origin with the Golgi apparatus and the endoplasmic reticulum of insects (Fig. 1b, the location of vesicles in the cell, the lower part of the Figure depicts one such vacuole) (Cho et al., 2011). It is suggested that the polar proteins Van Gogh/ Strabismus and Scribble can be responsible for positioning of such vacuoles close to the site of membrane synthesis (Cho et al., 2011). Wolbachia interacts with the cytoskeleton of the host’s cell to maintain the integrity and stability of vacuoles, similar to how the bacterial pathogens utilize such vacuoles to defend themselves against the host’s immune system (Ferree et al., 2005; Kumar, Valdivia, 2009; Creasey, Isberg, 2014).

Wolbachia requires a supply of many metabolites from the host (Jiménez et al., 2019; Newton, Rice, 2020). It has been hypothesized that the wMel strain native to D. melanogaster is dependent on the host for alanine, glycine, and serine metabolism, as well as lipopolysaccharide and biotin production (Jiménez et al., 2019; Newton, Rice, 2020). Wolbachia is completely dependent on the host for iron supply (Gill et al., 2014; Jiménez et al., 2019). On the other hand, dependence on substances supplied by Wolbachia has been shown for some insect species. For example, the bedbug Cimex lectularius utilizes riboflavin (Moriyama et al., 2015) and biotin (Nikoh et al., 2014) provided by the bacterium.

Among the key mechanisms of Wolbachia-host interaction is its impact on the cytoskeleton of host cells. Interaction with dynein and kinesin of host cell microtubules ensures Wolbachia’s passage into oocytes and hence its spread to the next generation (Ferree et al., 2005). Wolbachia is also reliant on clathrin/dynein-dependent capture by host cells for transport from somatic cell to germ cell (White et al., 2017).

Spontaneous loss of Wolbachia is sometimes reported, which can be explained by the response of the host’s immune system to the bacterium. Damaged organelles (for example, mitochondria) pose a threat to the cell. When such damage is detected, the organelle is eliminated by selective autophagy. This mechanism has recently been shown to be applicable to Wolbachia (Hargitai et al., 2022). Lysosome-mediated degradation of vacuoles containing Wolbachia may be a major cause of the host curing itself. Aging has been shown to decrease the efficiency of Wolbachia removal from the cells, resulting in Wolbachia actively proliferating and increasing its density in the host cells (Hargitai et al., 2022). Based on the obtained data, the authors conclude that autophagy may be a mechanism for controlling Wolbachia virulence.

It is logical to assume that if endosymbionts are observed in many generations of the same hosts, the host immune response to that organism is reduced. Since Wolbachia is the most common symbiont of invertebrates, it is likely that these bacteria have evolved an effective mechanism of protection against the host’s immunity, which only occasionally fails. It has been hypothesized that a new acquisition of Wolbachia infection triggers an immune response and oxidative stress in the host, whereas if there is evidence of a long time of symbiosis with a particular strain (a stable association of a strain of bacterium and a particular insect population), infection is not associated with oxidative stress (Zug, Hammerstein, 2015).

## Transcriptome analysis studies dedicated to the interactions in the Wolbachia–host system

Current approaches to determining the links between Wolbachia and the host rely on sequencing analysis. It is important to interpret the data from the studies of Wolbachia strain genomes in tandem with the results of host transcriptome studies.

Transcriptome analysis of the D. melanogaster lines infected with Wolbachia, equally with genomic studies, may shed light on the molecular mechanisms of interaction between these parts of the system. However, this method has drawbacks that have been repeatedly emphasized in the conducted studies. The host’s material is always in a larger quantity than material from the endosymbiont. To get around this limitation, it would make sense to use not the whole insect, but only the organs that have a higher density of this bacterium. The reproductive organs of the insect are suitable for this requirement, and appropriate studies have been made: on the ovaries (He et al., 2019; Frantz et al., 2023) and on the testes (He et al., 2019; Detcharoen et al., 2021). However, differences in gene expression levels between independent samples of the same type (one line infected with one strain) are often as significant as differences in gene expression levels between different types of samples (Detcharoen et al., 2021). This is most likely due to the contribution of other factors, such as unstable external conditions at the time of RNA extraction

The transcriptome in Wolbachia-infected D. melanogaster has also been analyzed using virgin and fertilized females (Detcharoen et al., 2021; Lindsey et al., 2021; Gruntenko et al., 2023), embryos (Mateos et al., 2019). However, the latter work found no significant differences in mRNA makeup between Wolbachia-infected and uninfected embryos (Mateos et al., 2019), which can probably also be explained by the contribution of other factors

Despite these drawbacks of using transcriptome analysis to study the influence of Wolbachia, it has been able to provide meaningful results concerning different aspects of the Wolbachia–Drosophila interaction. Further on we review several studies conducted over the last five years.

In a study investigating the phenomenon of CI and its mechanisms, first the ovarian transcriptome and then the testes transcriptome of adult D. melanogaster were analyzed (He et al., 2019). Comparisons were made between the transcriptomes of uninfected insects and those infected with the wMel strain. The authors identified the following functional groups of genes that are potentially susceptible to Wolbachia: “metabolism”, “transport”, “oxidation-reduction processes”, “immunity” and “individual development”. The authors hypothesize that Wolbachia is responsible for the regulation of the transcription in the opposite directions of a number of genes in female and male Drosophila. According to this hypothesis, when infected males mate with uninfected females, the resulting embryos have an imbalance in the levels of fertility restoration components, causing a cytoplasmic incompatibility effect (He et al., 2019). This popular hypothesis of the origin of CI is called titration-restitution model (Poinsot et al., 2003).

Another group of researchers also obtained transcriptome data on the topic of cytoplasmic incompatibility. A study was conducted to investigate the effect of various endosymbiotic bacteria on the transcriptome of early D. melanogaster embryos, but the authors found no effect of the Wolbachia wMel strain used in the study on the host transcriptome (Mateos et al., 2019). The authors concluded that the wMel strain does not alter maternal transcripts and does not lead to their degradation (Mateos et al., 2019).

There was a study of Wolbachia’s influence on D. melanogaster lines with different genotypes (Frantz et al., 2023). The authors studied ovarian transcriptomes of eight lines of D. melanogaster: four genetically diverse lines carrying one genotype of Wolbachia and derivatives of these lines that were cured of Wolbachia by tetracycline treatment. The host’s line genotype turned out to be a more significant factor affecting the transcriptome of the lines studied than the presence or absence of Wolbachia in them. However, the authors were still able to detect Wolbachia-induced differences in the expression of host genes involved in pathways related to cell cycle checkpoints, translation and metabolism, as well as cell division and recombination processes (Frantz et al., 2023).

The study conducted on the testes of two Drosophila species was aimed at investigating differences in the effect of the wMel strain on the native host species (D. melanogaster) and on a novel host species (D. nigrosparsa) to which the indicated strain was introduced by artificial transinfection of Wolbachia (Detcharoen et al., 2021). The detected differences affected such groups of orthologous genes as “oxidation-reduction processes”, “iron ions binding”, “activity of voltage-gated potassium channels” (Detcharoen et al., 2021).

In order to investigate the mechanisms of antiviral protection of host insects provided by Wolbachia, the transcriptomes of D. melanogaster flies infected with the Wolbachia wMel2 strain were analyzed (Lindsey et al., 2021). Two factors were simultaneously taken into account in the experimental design: Wolbachia infection or its absence, and Sindbis virus (SINV) infection or its absence. Four groups of insects (all possible combinations of these two factors) were acquired.

As a result of this analysis, the authors identified the following functional groups of genes that are potentially susceptible to Wolbachia: “stress response”, “RNA binding and processing”, “metabolism”, “ubiquitination”, and “transcription and translation”. The authors were unable to identify specific genes, the expression level of which would change as a result of the interaction between Wolbachia and virus. However, they constructed one core gene network linking genes responding to Wolbachia, genes responding to viruses, and genes, the response of which was induced by the combined effect of Wolbachia and the virus. Only genes attributed to the “metabolism” group (mainly amino acid metabolism and purine biosynthesis) got included in this network. The authors suggested that the discovered effect of Wolbachia on the synthesis of host nucleotides may be the reason for the suppression of virus replication (Lindsey et al., 2021).

In the study of the positive effect of the Wolbachia wMel- Plus strain on stress resistance of D. melanogaster flies, the transcriptomes of adult females of three lines of flies with the same nuclear genotype but differing in infection status (uninfected, infected with the wMelPlus strain, infected with the wMelCS112 strain) were compared (Gruntenko et al., 2023). Both Wolbachia strains induced changes in the expression levels of genes that belong to the functional groups “transmembrane transport”, “proteolysis”, “carbohydrate transport and metabolism”, “oxidation-reduction processes”, “regulation of alkaline phosphatase activity”, “embryogenesis”, and “stress response”. Nevertheless, the groups’ composition of differentially expressed genes partially differed between fly lines infected with different strains of Wolbachia (a pairwise comparison of the transcriptomes of infected fly lines against the transcriptomes of uninfected ones was conducted). The main difference in the expression of stress response genes was an increase in the level of transcription of the corazonin receptor (CrzR) gene in flies infected with the wMelPlus strain. Differences were also found between fly lines infected with different Wolbachia strains in the expression of different genes of alkaline phosphatases (which play a role in the stress response, participate in the dopamine synthesis cascade) (Gruntenko et al., 2023).

To summarize the above-mentioned studies, it can be concluded that Wolbachia affects the expression of hundreds of genes in flies of the genus Drosophila. These changes affect a multitude of processes that are combined into functional groups of the genes involved, the list of which differs in a number of studies. In turn, the functional groups can be matched to known Wolbachia effects that influence the observed host phenotype. The results of these large-scale transcriptome studies of Wolbachia-infected insects may help to guide more pinpointed experiments to specify the mechanisms in the Wolbachia–host system in the future.

With the development of sequencing technologies, new tools have become available. CappableSeq has been used to assemble the Wolbachia transcriptome of nematodes (Luck et al., 2017). This method could also be very promising for the study of insect Wolbachia transcriptomes, but no results of such an analysis have been published yet.

However, it is difficult to move from the results of specific studies to more global conclusions. Comprehensive analysis of data compiled from several experiments is known as metaanalysis. This direction of scientific search may in the future prove to be the most promising in studying the influence of Wolbachia on the host transcriptome.

## Conclusion

The Wolbachia–host system is very stable. Wolbachia evolved together with host species, and was also one of the factors directing their evolution. This mutualistic relationship is so deep and ancient that Wolbachia is compared to cell organelles located in the cytoplasm, such as mitochondria and chloroplasts. And even though a huge amount of information has been accumulated in this area, much is still unknown concerning the mechanisms maintaining this system.

This area of biology still lacks a systematization of knowledge that would not give rise to contradictions, beginning with the systematics of the genus and ending with the schematization of the molecular mechanisms of its effects. Wolbachia has acquired a controversial reputation, acting as a parasitic organism in some cases and as a mutualistic symbiont in others. A hundred years of studying this object does not provide a complete picture.

Since Wolbachia has become famous for manipulating the host’s reproductive strategy, most studies are devoted to this topic, and not enough attention is paid to another important area – the Wolbachia influence on the processes occurring in somatic cells. Wolbachia not only affects reproduction but other vital signs in the host as well. It is necessary to continue investigation of less popular and well-studied aspects of the Wolbachia–host interactions using new bioinformatics methods and technologies that allow for fundamentally new experiments. The application of these approaches has already contributed to significant progress in the area, but the development of ideas concerning the relationship between insects and the endosymbiotic bacterium W. pipientis is not yet complete.

## Conflict of interest

The authors declare no conflict of interest.
